# Assessing the impact of topography on malaria exposure and malaria epidemic sensitivity in the Western Kenya highlands

**DOI:** 10.1186/1475-2875-9-S2-P59

**Published:** 2010-10-20

**Authors:** Christine L Wanjala, Andrew K Githeko, John N Waitumbi

**Affiliations:** 1 Climate and Human Health Research Unit, Centre for Global Health Research, Kenya Medical Research Institute, P. O. Box 1578, Kisumu 40100, Kenya; 2 Egerton University, Nakuru, Kenya; 3 Kenya Medical Research Institute/Walter Reed Project, Kisumu, Kenya

## Background information

Malaria in the Western Kenya Highland is characterized by unstable and high transmission variability which results into epidemics during periods of suitable climatic conditions. The sensitivity of a site to malaria epidemics depends on the level of immunity of human population. This study examined how terrain in the highlands affects the exposure and sensitivity of a site to malaria.

## Methodology

The study was conducted in five sites in western Kenya highlands, two U-shaped valleys (Iguhu, Emutete), two V-shaped valleys (Marani, Fort-Ternan) and one plateau (Shikondi) for twelve months among 6-15 years old children. Exposure to malaria was tested using circum-sporozoite protein and merozoite surface protein immunochromatographic antibody test; malaria infection was tested by microscopic examination of thick and thin smears, the children's homes were georeferenced using global positioning system and the data used for mapping the study sites Results The mean antibody prevalence was 20.5% in Iguhu, 23.6% in Emutete, 12.7% in Shikondi, 9.6% in Fort-Ternan and 10.6% in Marani. The mean malaria infection prevalence was 23.5% in Iguhu, 21.1% in Emutete, 5.1% in Shikondi, 3.1% in Fort-Ternan and 3.6% in Marani. There was a significant difference in the antibodies and malaria infection prevalence among the two valley systems and the plateau (P<0.05). There was no significant difference in the antibodies and malaria infection prevalence within the U-shaped valleys and within the V-shaped valleys (P> 0.05). There was a 5-fold and a 2-fold greater parasites and antibody prevalence respectively, in the U-shaped compared to the V-shaped valleys. The plateau antibody and parasite prevalence was similar to that of the V-shaped valleys. There was clustering of malaria antibodies and infections around the swamps in the U-shaped, the infections were randomly distributed in the V-shaped valleys and less clustered at the plateau at low altitudes.

## Conclusion

The findings of this study indicate that drainage characteristics of the valleys systems affect the exposure of the human population to malaria parasites and the immune response to malaria. The spatial distribution maps showed clustering of malaria infections around the swamps therefore topography maps can be reliably used to identify the affected areas and the scarce resources focused to these areas to control malaria.

